# ASSOCIATION BETWEEN FRAILTY AND FIVE-YEAR MORTALITY AFTER HIP FRACTURE SURGERY IN OLDER PATIENTS

**DOI:** 10.1093/geroni/igad104.3189

**Published:** 2023-12-21

**Authors:** Jung-Yeon Choi, Kwang-il Kim

**Affiliations:** Seoul National University Bundang Hospital, Seoul, Republic of Korea; Seoul National University Bundang Hospital, Seoul, Republic of Korea

## Abstract

We aimed to identify the association between frailty and 5-year mortality after hip fracture surgery. The Hip-Multidimensional Frailty Score (Hip-MFS) was calculated using the Comprehensive Geriatric Assessment (CGA). The primary outcome was 5-year mortality. Among 1,363 patients who underwent HF surgery, 598 (44%) patients underwent CGA, and 536 patients were included in the final analysis. The mean age was 80.5 years and 71.3% were females. A total of 223 (41.6%) patients experienced postoperative complications. The median observation time was 1999.5 days, and the overall mortality rate was 60.4% (n = 324), whereas the 1-year mortality and the 5-year mortality rate after HF surgery were 13.8% (n = 74) and 42.8% (n = 235), respectively. In the multivariate regression analysis, after accounting for clinical and demographic factors, the high-risk Hip-MFS group and the group with postoperative complications had hazard ratios of 1.49 (95% confidence interval [CI] 1.090–2.037, p = 0.012) and 1.498 (95% CI 1.139–1.970, p = 0.004), respectively. Patients with postoperative complications with low Hip-MFS showed better 5-year survival than those without postoperative complications with high hip-MFS in the Kaplan-Meier curve (p = 0.013). Compared with the occurrence of postoperative complications, the frailty status evaluated with the Hip-MFS had a more significant impact on long-term mortality after hip fracture surgery.

